# The complete mitochondria genome sequence of *Heortia vitessoides* Moore

**DOI:** 10.1080/23802359.2019.1687348

**Published:** 2019-11-13

**Authors:** Yonglin Liao, Miaofeng Xu, Jihua Wang

**Affiliations:** a Crops Research Institute, Guangdong Academy of Agricultural Sciences, Guangdong Key Laboratory for Crops Genetic Improvement, Guangzhou, China;; b Institute of Plant Protection, Guangdong Academy of Agricultural Science, Guangdong Provincial Key Laboratory High Technology for Plant Protection, Guangzhou, China;; c Technical Center of Gongbei Customs District, Zhuhai, China

**Keywords:** *Heortia vitessoides* (Moore), mitochondria genome, phylogenetic relationship

## Abstract

*Heortia vitessoides* (Moore) is the most destructive defoliating pests in *Aquilaria sinensis* (Loureiro) Sprenger forests in Southern China. The complete sequences of mitochondria is reported: a circular molecule of 15,516 bp in size included 40.13% for A, 40.79% for T, 11.23% for C and 7.86% for G. There are 60 genes including 3 species with 12 protein-coding genes, 2 different species ribosomal RNA genes (S and L rRNA species), 46 transfer RNA genes (20 RNA species). *H. vitessoides* (Moore) and other 19 species belonging to lepidopteran were phylogenetic and analyzed by MEGA 6.06 with neighbor-joining methods. The mtDNA of *H. vitessoides* (Moore) were clustered in lepidopteran superfamilies.

*Heortia vitessoides* (Moore) belonging to Lepidoptera is one of the specialists defoliating pest affecting *A. sinensis* forests in southern China (Qiao et al., 2016). The sequences of mtDNA were the easiest to isolate and characterize the gene content, which could be established readily and be available for 4 lepidopteran superfamilies: Tortricoidea (Adoxophyes); Pyraloidea (Ostrinia); Bombycoidea (Bombyx, Antheraea) and Papilionoidea (Coreana) (Brown et al. 1994; Cameron and Whiting 2008). In this study, the complete mitochondria genome of *H. vitessoides* was sequenced and cluster analysis was constructed using the neighbor-joining method.

*Heortia vitessoides* were collected from the Fengguang Specialized Cooperative plantation (N21°47′21.37″, E111°14′18.46″), Dianbai, Guangdong, China, on 11 April 2019. The heads of *H. vitessoides* were cut and collected for total genomic DNA isolation (accession number: Hdb1), which was stored in Guangdong Key Laboratory for Crops Genetic Improvement, Guangzhou. The mtDNA （Hdb1）of *H. vitessoides* was sequenced using Illumina Hiseq 2500 and assembled with *Chilo sacchariphagus* (Genebank accession NC_029716) as a reference by MITObim v1.9 (https://github.com/chrishah/MITObim) (Hahn et al. 2013). The cpDNA Genome annotation was conducted in Geneious R10 (Biomatters Ltd., Auckland New Zealand) and aligned phylogenetically with related species, and the physical map was drawn with the web-based tool OGDRAW (http://ogdraw.mpimp-golm.mpg.de) (Lohse et al. 2013).

The circular cpDNA of *H. vitessoides* (Genebank accession No: MN385683) is 15,516 bp in length, which included 40.13% for A, 40.79% for T, 11.23% for C and 7.86% for G. There are 60 genes in the mtDNA, including 3 species with 12 protein-coding genes, 2 different species ribosomal RNA genes (S and L rRNA species), 46 transfer RNA genes (20 RNA species, tRNA-Lys, tRNA-Asn, tRNA-Met, tRNA-Leu, tRNA-Ile, tRNA-Cys, tRNA-Thr, tRNA-Tyr, tRNA-Asp, tRNA-Phe, tRNA-Val, tRNA-Arg, tRNA-Glu, tRNA-Ala, tRNA-Pro, tRNA-Gly, tRNA-Gln, tRNA-Trp, tRNA-His, tRNA-Ser). There are 14 genes (ND1, ND4L, ND3, ATP6, ND2, ND4, ND5, COX1, CYTB, COX2, ND6, COX3, S-rRNA and L-rRNA). Most of the genes were multi-copies, Ile had 7 copies, which was the highest. Based on the 14 protein-coding gene sequences of *H. vitessoides*, phylogenetic analysis with other 19 species belonging to lepidopteran were constructed using MEGA 6.06 (Tamura et al. 2013). The phylogenetic tree was deconstructed using the neighbor-joining method (NJ) which showed that *H. vitessoides* was clustered in Pryalidae (Figure 1).

**Figure 1.**
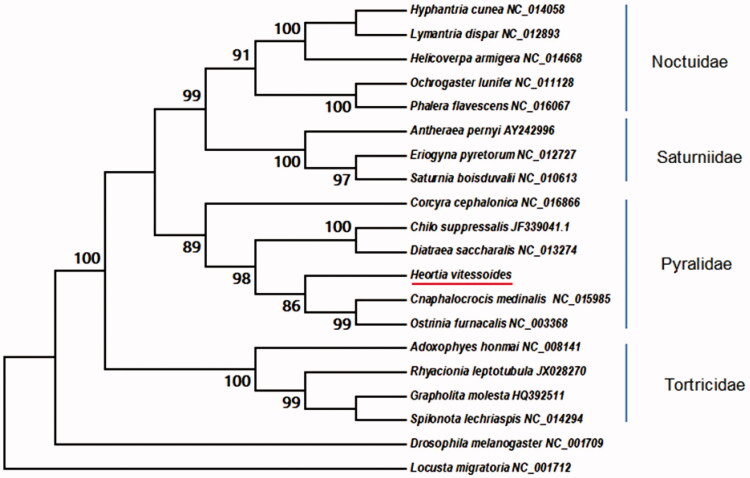
The ML The phylogenetic tree of the *H. vitessoides* and similar families based on mtDNA sequence. Numbers labeled beside the node are bootstrap support values. *H. vitessoides* was the most similar with *C. medinalis* and *O. furnacalis*.
